# Gum Arabic protects the rat heart from ischemia/reperfusion injury through anti-inflammatory and antioxidant pathways

**DOI:** 10.1038/s41598-022-22097-0

**Published:** 2022-10-14

**Authors:** Eman Gouda, Fawzi Babiker

**Affiliations:** Department of Physiology, Faculty of Medicine, Health Science Center, Kuwait University, Safat, P.O. Box 24923, 13110 Jabriya, Kuwait

**Keywords:** Biochemistry, Physiology, Cardiology

## Abstract

Gum Arabic (GA) is a plant exudate with antioxidant and anti-inflammatory effects. GA has shown promise in protection from and treatment of kidney failure, however, its role in the protection of the heart from ischemia and reperfusion (I/R) has not been investigated. This study investigated the antioxidant and anti-inflammatory effects of Gum Arabic (GA) in the protection of the heart against ischemia/reperfusion (I/R) injury. Langendorff-perfused Wistar rat hearts were divided into seven groups. One group which was subjected to I/R with no other treatment served as the control group. The second group was subjected to buffer perfusion with no ischemia (sham group). The third group was perfused with GA in the absence of ischemia (sham + GA). The rest of the hearts were isolated from rats that had been treated with GA for 4 or 2 weeks in the drinking water, or GA that had been infused intravenously 2 h before sacrifice or added to perfusion buffer at reperfusion. Hemodynamics data were digitally computed; infarct size was measured using 2,3,5-triphenyltetrazolium chloride (TTC) staining and cardiomyocyte injury was assessed by quantifying creatine kinase (CK) and lactate dehydrogenase (LDH) enzymes. The total oxidants (TOS) and antioxidants (TAS), superoxide dismutase (SOD) and pro- and anti-inflammatory cytokines levels were estimated by ELISA. GA treatment for 2 weeks, 4 weeks or 2 hours before sacrifice resulted in a significant (P < 0.05) improvement in cardiac hemodynamics and reduction in infarct size and cardiac enzyme levels compared to respective controls. However, GA administration at the time of reperfusion did not protect the hearts against I/R injury. Furthermore, GA treatment decreased the pro-inflammatory and anti-inflammatory cytokines levels. The levels of TOS in the effluent were significantly decreased (P < 0.05) and SOD levels were significantly (P < 0.05) increased by GA administration. GA protected the heart against I/R injury when administered for 2 or 4 weeks or when infused 2 hours before sacrifice. GA treatment decreased the total oxidants levels, the pro-inflammatory cytokines TNF-α, IL-1β and IL-6 protein levels and increases SOD and anti-inflammatory cytokine IL-10 protein levels.

## Introduction

Ischemic heart disease (IHD) is a leading cause of morbidity and mortality worldwide^1^. It negatively affects coronary perfusion, which diminishes the supply of oxygen and essential nutrients to cardiac myocytes^2^. The reduced oxygen supply impairs mitochondrial oxidative phosphorylation, thus depleting the immediate source of ATP in cardiac myocytes leading to myocardial infarction (MI), which ultimately compromises cardiac function^3^. Myocardial infarction provokes cellular adaptations and remodeling of viable tissues, including increased interstitial connective tissue, compensatory cellular hypertrophy and coronary revascularization^2^. Remodeling of the heart coupled with a reduced number of functioning myocytes might cause contractile dysfunction in viable cardiomyocytes, arrhythmia and left heart failure^4^.

The role of reactive oxygen species (ROS) and inflammatory cytokines and their interactions with other signaling molecules in ischemia/reperfusion (I/R) injury is not completely understood. Although the role of oxidative stress in I/R injury and postconditioning protection to the heart is well documented^5,6^, the molecular mechanisms underlying this phenomenon are enigmatic^7^. ROS damage cellular proteins, lipids, carbohydrates and DNA through multiple mechanisms, which in the case of the heart, cause I/R injury and IHD^8^. On the other hand, antioxidants protect the organ systems from excessive free radicals and produce a neutralized *milieu* by decreasing ROS levels^8^. During I/R injury, the balance between oxidants and antioxidants in organs gets adversely affected either due to excessive ROS production at the beginning of reperfusion or a decrease in antioxidants levels in the case of comorbidities such as diabetes mellitus^9^. However, higher levels of antioxidants in the blood may counteract the effects of excessive free radicals, thereby modulating the extent of I/R injury.

Inflammatory cytokines play essential roles in health and diseases^10,11^. Pro-inflammatory cytokines such as tumor necrosis factor-alpha (TNF-α), interleukin-1-beta (IL-1β), and IL-6 provoke and exacerbate the inflammation^10^. On the other hand, anti-inflammatory cytokines play an essential role in counterbalancing the influence of pro-inflammatory cytokines^10,11^. There exists a natural balance between pro- and anti-inflammatory cytokines, which could be disrupted by stress or a disease^10,11^. The potent anti-inflammatory cytokine, IL-10, suppresses the inflammatory responses generated by TNF-α, IL-1β, and IL-6^11^.

Although several chemotherapeutic agents coupled with invasive heart protection procedures are being continuously used to protect the heart from I/R injury, the roles of naturally available compounds have not been thoroughly evaluated. Gum Arabic (GA) is a naturally dried exudate extracted from Acacia trees, *Acacia Senegal* and *Acacia seyal*^12^. GA was approved by the United States Food and Drug Administration (FDA) as a food additive in 1969^13^. Since then, it is being widely used as a stabilizer, emulsifier, and a thickening material in the food industry^14^. It is also used as an enhancer of the physical properties of medicines^14^ and a component of adhesive hydrogels used in wound healing^15^.

Furthermore, GA possesses anti-inflammatory^16^ and antioxidant properties^16^, therefore, it could be used in the treatment of inflammation^16^, and it apparently, delays the progression of chronic renal failure^17^. To date, most of the studies on GA have investigated its effects on renal function and renal failure. GA imparts beneficial effects on kidney function and protects the kidneys from I/R injury^18^. This study investigated the potential protective effects of GA in the heart against I/R injury and its impact on oxidative stress and the levels of inflammatory cytokines during ischemia and reperfusion.

## Materials and methods

### Experimental animals

Male Wistar rats (250–350 g) were obtained from the Animal Resources Center at Kuwait University. The study was approved by the Health Science Center, Kuwait University Animal Ethics Committee. The study was carried out following the EU Directive 2010/63/EU for the use of animals in experimental research. All rats were maintained under controlled temperature (21–24 °C), 12 h light/dark cycle (7 a.m.–7 p.m.), and humidity (50%). The rats were housed in plastic cages (2 rats/cage), and food and water were available ad libitum. The rats were anesthetized with an intraperitoneal injection of sodium pentobarbital (60 mg/kg) and anticoagulated with an intraperitoneal injection of heparin (1000 U/kg body weight). The drug infusion 2 hours before animal sacrifice was done under general anesthesia. The rats were killed by cervical dislocation under general anesthesia. The diaphragm was cut carefully to expose the thoracic cavity. A bilateral incision opened the thorax along the lower margin of the chest from the last to the first rib. The thoracic cage was folded back, and the heart was exposed and excised. The heart was immediately immersed in cold (4 °C) Krebs–Hensleit (KH) buffer to minimize ischemic injury that might be inflicted during the time between excision and restoration of vascular perfusion^19^.

### Drugs and chemicals

**Figure 1:**
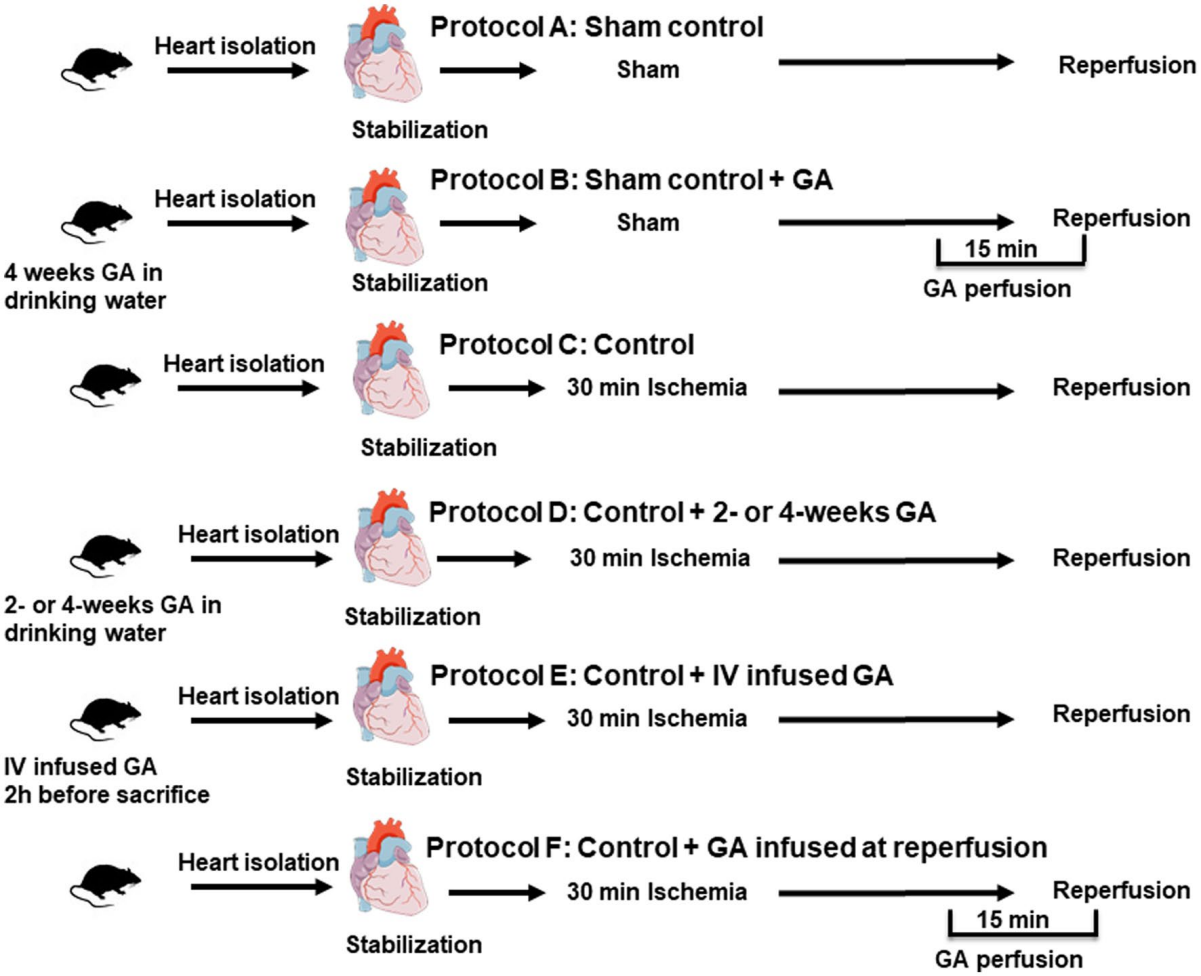
Schematic representation showing the experimental protocols used in the study (n = 8). A: vehicle treated sham controls. B: GA treated sham controls. C: unprotected ischemia–reperfusion control (Ctr). D: protection with 2 or 4 weeks GA in the drinking water, E: IV infused GA, F: GA infused with the perfusion buffer 5 min before reperfusoin and continued for 10 min after the start of reperfusion. *GA* Gum Arabic, *IV* intravenous injection.

The study included seven groups (n = 8) (Fig. 1). In group one, the hearts were isolated from control rats and subjected to I/R protocol without tying the thread to produce ischemia (sham) (Fig. 1 protocol A). The second group of hearts was isolated from rats administered with 50 g GA dissolved in 1L distilled water (50 g/1L) and treated similar to the sham group (sham + GA) (Fig. 1, protocol B). The third ischemic control group included hearts subjected to I/R with no additional treatment (Ctr) (Fig. 1, protocol C). The rest of the rats were divided into two major groups. The first major group received long-term treatment of 50 g/L GA and was divided into two subgroups; one subgroup was administered with GA for 2 weeks, and the other one was administered with GA for four weeks as described previously^20^ (Fig. 1, Protocol D). The second major group comprised GA acute treatment subgroups. One subgroup was injected intravenously with 0.2 g/kg GA dissolved in normal saline 2 hours before the heart isolation (Fig. 1, protocol E). The hearts isolated from the other subgroup were perfused with 2 g of GA dissolved in 1 L of KH solution starting 5 min before the reperfusion and continued for 10 min during reperfusion^21^ (Fig. 1, protocol F). The hearts isolated from the two major groups of rats were subjected to 30 min ischemia followed by 30 min reperfusion. All GA doses where adopted from previous studies in kidney diseases^20,21^.

### Experimental procedure

Heart cannulation and perfusion were performed using a modified Langendorff setup for rat heart perfusion as described previously^22^. Briefly, the isolated heart was immersed in cold KH buffer (4 °C), immediately mounted and fixed to a small cannula, and then perfused retrograde with freshly prepared KH buffer. The buffer was gassed with a mixture of O_2_ (95%) and CO_2_ (5%). The pH of the perfusion buffer was 7.35–7.45, and its temperature was maintained at 37 °C by circulation in a temperature controller using a water bath (RMS Lauda, Dr. R. Wobser GmbH & Co, Germany) and a Techno Circulator (Cole-Parmer Instrument Company, USA). The heat temperature was monitored by a needle thermistor probe (Thermalert TH-5, Physitemp, USA) inserted at the apex of the heart. The heart was allowed 10 min for acclimatization until the normal contractile function and the heart rhythm were restored. A water-filled latex balloon was placed and secured in the left ventricular (LV) cavity^23^. The balloon was attached to a pressure transducer and a DC bridge amplifier (DC-BA) with a pressure module (DC-BA type 660, Hugo-Sachs Electronik, Germany) and interfaced to a personal computer for monitoring of the hemodynamics. The balloon was inflated using a microsyringe until a baseline left ventricular end-diastolic pressure (LVEDP) of 6 mmHg was reached. The left ventricular developed pressure (DPmax, mmHg) was derived from the acquisition of the left ventricular end-systolic pressure (LVESP) using the Max–Min module (Number MMM type 668, Hugo-Sachs Elektronik-Harvard Apparatus GmbH, Germany), which converts the output from a DC bridge amplifier to DPmax by subtracting LVEDP from the maximal LVESP.

The coronary vascular dynamics were evaluated by assessing the coronary flow (CF, mL/min) and coronary vascular resistance (CVR, mmHg/mL/min). The CF was continuously measured using an electromagnetic flow probe attached to the inflow of the aortic cannula and interfaced to a personal computer. Continuous monitoring of the CF in mL/min was verified manually by a timed collection of the coronary effluent. The CVR was computed once every 10 s along with the hemodynamics data by an online data acquisition program (Isoheart software V 1.524-S, Hugo-Sachs Electronik, Germany). In all protocols, the perfusion pressure (PP) was kept constant at 50 mmHg throughout the experimental procedures. The PP was measured immediately downstream from the flow probe in a branch of the aortic cannula using a Statham pressure transducer (P23 Db). Constant PP was ensured using an electronic perfusion assembly (Module PPCM type 671, Hugo-Sachs Electronik, Germany).

The heart was then instrumented with pacing electrodes placed on the right atrial (RA) appendage. Regional ischemia was produced by passing a suture around the left coronary artery approximately 0.5 cm below the atrioventricular groove. A rigid plastic tube was positioned between the heart and the suture to ensure complete occlusion of the coronary vessel and allow the re-opening of the occluded vessel without any mechanical damage. All hearts were subjected to 30 min ischemia followed by 30 min reperfusion. In sham groups, the suture was passed around the left coronary artery without producing ischemia.

### Sample collection and storage

Coronary effluent was collected manually at the apex of the heart from the coronary outflow in small tubes at the end of the reperfusion phase. The hearts were also collected at the end of reperfusion. All heart samples and coronary effluents were frozen in liquid nitrogen and stored at − 80 °C for further analysis.

### Tissue homogenization and prtein extraction

Snap-frozen heart tissue (n = 4) was homogenized in RIPA buffer (the buffer contained Tris, NaCl, NP-40, EDTA, Na-deoxycholate, 0.1% SDS, protease inhibitor (Sigma Aldrich, St. Louis, Missouri, cat#s8820)), benzamidine and phenylmethane sulfonyl fluoride or phenylmethylsulfonyl fluoride (PMSF). Na-deoxycholate, protease inhibitor and PMSF were added on the day of the experiment)) as described in the assay procedure. The homogenate was centrifuged at 15,000×*g* for 25 min at 4 °C, and the supernatant was separated and stored at − 80 °C.

### Evaluation of infarct size by triphenyltetrazolium chloride (TTC) staining

The infarct size was measured blindly using Scion ImageJ (ImageJ, Wayne Rasb and National Institute of Health, USA; n = 4/group). At the end of the reperfusion, the hearts were collected and stored overnight at − 20 °C. The next day, each heart was sliced transversely into 4–6 pieces from apex to base. The slices were then incubated in 1% TTC solution in isotonic (pH 7.40) phosphate buffer and then fixed in 4% formaldehyde for 24 h. Red-stained viable and the pale, non-stained necrotic infarcted regions in heart sections were manually indicated on the image and measured by the software. The percentage of the infarcted area of the LV was calculated for every section of the heart.

### Assessment of heart injury via creatine kinase and lactate dehydrogenase levels

Cardiomyocyte injury was evaluated by quantifying creatine kinase (CK) and lactate dehydrogenase (LDH) levels in the coronary effluent at the end of the reperfusion period as described previously^24^.

### Estimation of cytokines by enzyme-linked immunosorbent assay (ELISA)

The heart homogenate (n = 4) was used for cytokines estimation The pro-inflammatory cytokines- TNF-α (cat# MBS175904), IL-1 (cat# MBS825017), and IL-6 (cat# MBS355410) and the anti-inflammatory cytokine, IL-10 (cat# MBS355232) were estimated in the homogenate of heart tissue using ELISA (n = 4/group).

### Estimation of total oxidant levels

The total oxidant levels (TOS) were quantified using the TOS Status Assay Kit (Rel Assay Diagnostics Kit, cat#RL035)^25^. The homogenized samples were placed on ice and diluted 1:10 with deionized water. Next, 18.75 µl of deionized water (blank), standard solution or homogenized samples were pipetted into the corresponding wells of a 96-well plate. Then, 125 µl of reagent 1 was added to all wells, and the initial absorbance was read at 530 nm after 30 s. Then, 6.25 µl of reagent (colored 2,2'-azino-bis (3-ethylbenzothiazoline-6-sulphonic acid (ABTS) radical solution) was added to all wells and incubated for 10 min at room temperature, and the second absorbance was measured at 530 nm. The results were calculated by fitting the absorbance values into a formula given in the assay manual.

### Estimation of total antioxidant levels

The heart homogenate (n = 4) was also used to analyze the total antioxidant status (TAS) after GA treatment, before I/R procedure or at reperfusion using a commercially available assay kit (Rel Assay Diagnostics kit, cat#RL032)^26^. Deionized water (7.5 µl; blank), standard solution or homogenized samples were pipetted into the corresponding wells of a 96-well plate. Then, 125 µl of reagent 1 was added to all wells, and the initial absorbance was read at 660 nm after 30 s. Next, 6.25 µl of reagent 2 (ABTS radical solution) was added to all wells and incubated for 10 min at room temperature, and the second absorbance was measured at 660 nm. The results were calculated by fitting the absorbance values into a formula given in the assay manual.

### Antioxidant enzyme superoxide dismutase status

Superoxide dismutase (SOD; EC.1.15.1.1) activity was measured by UV spectrophotometer with a commercially available assay kit (Cat. No. SD 125; Randox Labs Ltd., Crumlin, UK) according to the manufacturer’s instructions.

### Western blotting

Left ventricles (*n* = 4) homogenates were used for the evaluation of the protein levels. The levels of the proteins in question were determined as described previously (Al-Herz et al.). Equal loading was checked by stripping the membrane and reprobing with GAPDH antibodies. Cleaved caspase-3 and SOD levels were determined using monoclonal antibodies (Cell Signaling Technology, Inc cat# 9662 and #37385 respectively). Detection was performed using enhanced chemiluminescence after incubation with a suitable secondary antibody conjugated to horseradish peroxidase (ECL; Cell Signaling Technology). The ratio of the target protein band density to total loaded protein was estimated. Detection was performed using enhanced chemiluminescence after incubation with a suitable secondary antibody conjugated to horseradish peroxidase (ECL; Cell Signaling Technology). Densitometric analysis was performed using Quantity One software (BioRad).

### Statistical analysis

The data were represented as the mean ± SEM. Two-way analysis of variance (ANOVA) was used to assess the significant differences between the means of different groups. The post hoc analysis using the Tukey test was conducted for further comparison. An unpaired two-sided ‘*t*’ test was used to analyze differences in infarct size between the groups. In all cases, P < 0.05 was considered statistically significant.

### Ethics statement

The study was approved by the Health Science Center, Kuwait University Animal Ethics Committee. The study was carried out following the EU Directive 2010/63/EU for the use of animals in experimental research. All methods are reported in accordance with ARRIVE guidelines.

## Results

### Involvement of GA in heart protection

**Figure 2:**
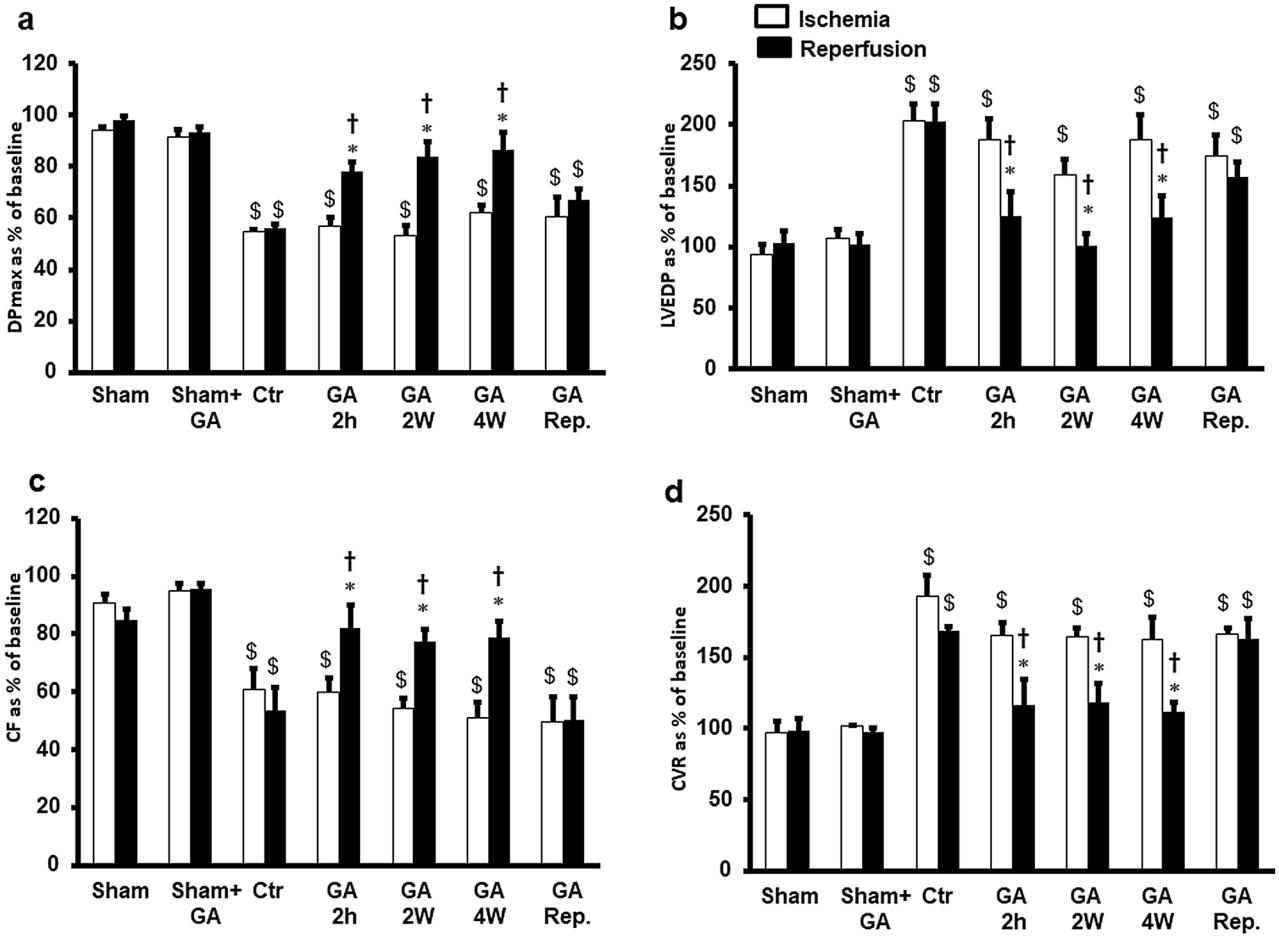
Left ventricle function (DPmax and LVEDP) and coronary vascular dynamics (CF and CVR) during post-ischemic recovery after GA treatment protocols (n = 8). The data were computed after 30 min reperfusion and expressed as the mean ± SEM. *DPmax* maximum developed pressure, *LVEDP* left ventricular end-diastolic pressure, *CF* coronary flow, *CVR* coronary vascular resistance, *Ctr* control, *Rep* reperfusion, *GA 2 h* Gum Arabic infusion 2 h before sacrifice, *GA 2 W* Gum Arabic administration for 2 weeks, *GA 4 W* Gum Arabic administration for 4 weeks, *GA Rep*. Gum Arabic infusion at reperfusion. *P < 0.05 compared to respective controls, ^†^P < 0.05 compared to the ischemic period and ^$^P < 0.05 compared to vehicle or GA treated sham controls.

The body and heart weights did not show significant differences between the experimental groups. The GA intake in experimental groups also did not show significant differences (42.57 ± 1.4 ml/rat in controls vs. 48.6 ± 2.03 ml/rat in GA treated animals). Induction of regional ischemia in hearts resulted in a significant (P < 0.05) deterioration in left ventricular and coronary arterial dynamics compared to sham and GA-treated sham rats (Fig. 2 and Table 1). Administration of GA for 4 weeks, 2 weeks or 2 hours to the rats before the heart isolation protected the heart against I/R injury compared to the results of the respective ischemic periods and the untreated controls. However, this protection was not observed when GA was infused at the start of reperfusion (Fig. 2). Treatment with GA for 4 weeks, 2 weeks or 2 hours before heart isolation significantly normalized the DPmax values compared to that in respective ischemic periods and the untreated controls (P < 0.01) (Fig. 2a). This treatment also improved LVEDP, reducing it significantly (P < 0.05) compared to that of the respective ischemic periods and untreated control groups (P < 0.05) (Fig. 2b). This improvement in DPmax and LVEDP was also seen in LV contractility, which was significantly (P < 0.05) normalized compared to that in the respective ischemic periods and untreated control groups (Table 1). Significant (P < 0.05) improvement was also produced in the coronary vascular dynamics. Ischemia deteriorated the CF but was significantly (P < 0.05) normalized to baseline values compared to that in the respective ischemic periods and untreated control groups (Fig. 2c). CVR was significantly (P < 0.01) decreased by these treatments (Fig. 2d). Acute treatment with GA at the time of reperfusion did not induce a significant difference either in the LV hemodynamics or in the coronary vascular dynamics compared to that in the respective ischemic periods and untreated control groups. There were no significant differences in left ventricular dynamics, contractility and coronary vascular dynamics between groups that had been treated with GA for 4 weeks, 2 weeks or 2 hours before the heart isolation in left ventricular and coronary vascular dynamics (Fig. 2, Table 1). Left ventricular dynamics, contractility and coronary vascular dynamics in groups that had been treated with GA for 4 weeks, 2 weeks or 2 hours before the heart isolation were not significantly different from those of sham and GA treated sham groups. There were also no significant differences in the parameters measured between the sham and GA treated sham controls (Fig. 2, Table 1).

**Table 1:** Effects of ischemia/reperfusion and GA treatment on heart contractility (n = 8).

Treatment	+ dP/dt		− dP/dt	
	Ischemia	Reperfusion	Ischemia	Reperfusion
Sham	91.76 ± 0.66	89.07 ± 1.68	91.86 ± 1.31	90.64 ± 0.67
Sham + GA	92.50 ± 0.61	91.11 ± 0.37*	93.83 ± 0.65	93.46 ± 1.82*
Ctr	66.41 ± 7.51^$^	59.85 ± 7.93^$^	63.10 ± 4.87^$^	62.14 ± 7.91^$^
GA 2 h	65.01 ± 4.42^$^	86.71 ± 3.65*	70.74 ± 2.75^$^	89.87 ± 4.86*
GA 2 W	68.23 ± 4.20^$^	85.18 ± 5.13*	64.76 ± 2.17^$^	86.37 ± 5.75*
GA 4 W	64.05 ± 6.26^$^	87.69 ± 3.39*	64.16 ± 4.79^$^	87.84 ± 4.62*
GA reperfusion	66.86 ± 9.73^$^	67.59 ± 8.37^$^	72.24 ± 5.66^$^	75.16 ± 5.79^$^

*Ctr* control, *GA 2 h* gum arabic infusion 2 h before sacrifice, + *dP/dt* and − *dP/dt* left ventricle contractility indices, *GA 2 W* gum arabic administration for 2 weeks, *GA 4 W* gum arabic administration for 4 weeks, *Rep* Reperfusion.

*P < 0.05 compared to control and ^$^P < 0.05 compared to sham and sham + GA.

### Variability in infarct area of hearts in control and experimental groups

**Figure 3:**
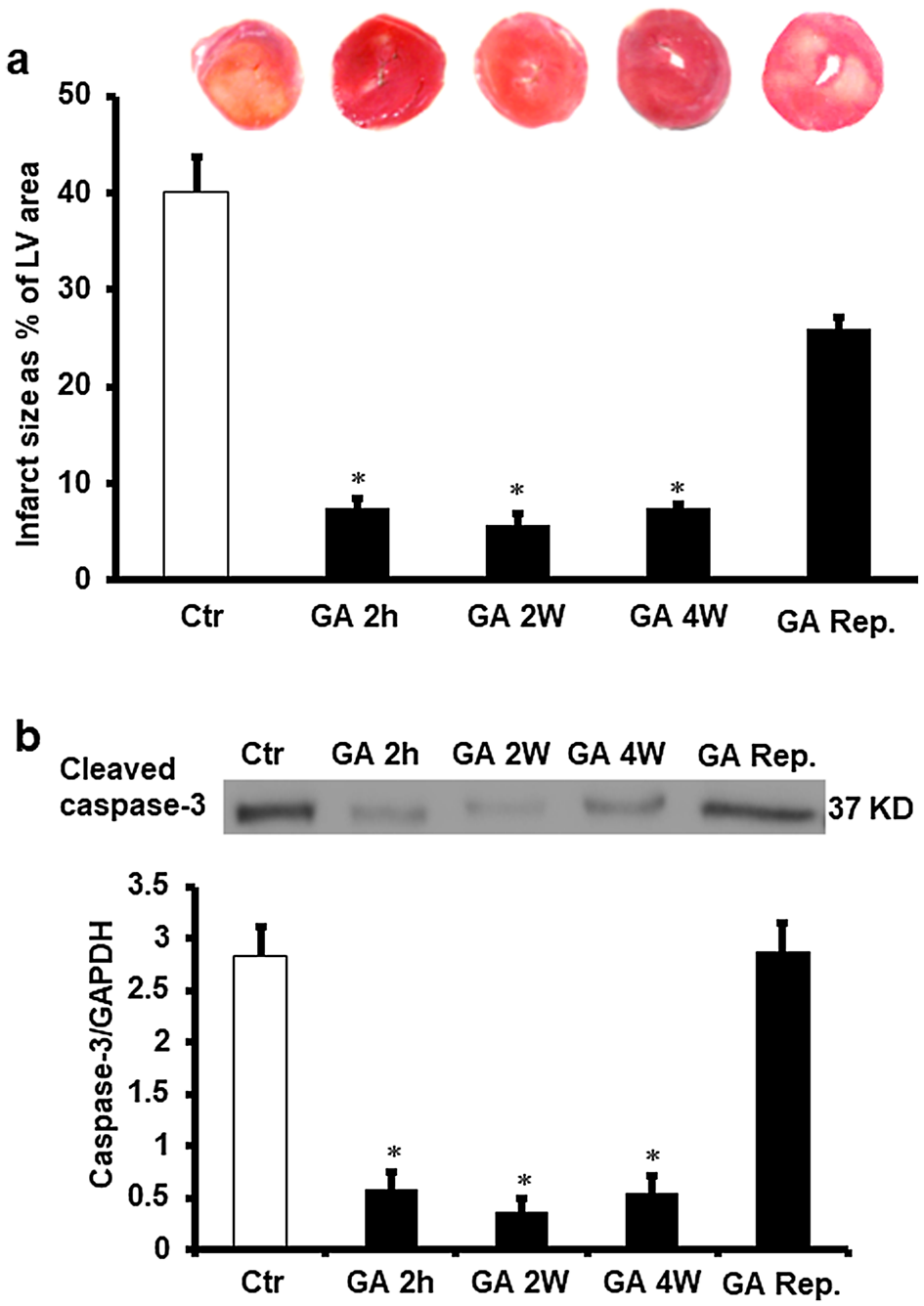
Evaluatin of infarct size and apoptosis markers. (**a**) Infarct size after GA treatment (n = 4). Top: representative 2,3,5-triphenyl-2H-tetrazolium chloride-stained heart slices for each treatment condition. Bottom: measured infarct size, normalized to the LV area, in isolated rat hearts at the end of reperfusion. (**b**) Cleaved caspase-3 protein levels after GA treatment (n = 4). Top: Western blot showing casepase-3 protein levels corrected to GAPDH. Bottom: Cleaved caspase-3 protein levels corrected to GAPDH. *Ctr* control, *Rep* reperfusion, *GA 2 h* Gum Arabic infusion 2 h before sacrifice, *GA 2 W* Gum Arabic administration for 2 weeks, *GA 4 W* Gum Arabic administration for 4 weeks, *GA Rep*. Gum Arabic infusion at reperfusion. *P < 0.001 compared to control. Full length gel was included in supplementary figure (Fig. 3S).

In line with the results of hemodynamics studies, the infarct size, presented as a percentage of the area of the left ventricle was significantly (P < 0.01) decreased by GA exposure for 4 weeks, 2 weeks or 2 h before the heart isolation compared to the values of the respective untreated controls. The protective effects were masked when GA was dosed at the beginning of reperfusion (Fig. 3a). There were no significant differences in the infarct size ratios between the groups with GA treatments for 4 weeks, 2 weeks or 2 hours before heart isolation (Fig. 3a). Similar results were obtained when cleaved caspase-3 protein levels (Fig. 3b) and heart enzyme levels (Table 2) were measured. Furthermore, the cardiac enzyme levels in controls were significantly (P < 0.001) higher than that of the sham and GA treated sham controls (Table 2).

**Table 2:** Effects of ischemia/reperfusion and GA treatment on cardiac enzymes levels (n = 8).

Treatment	CK IU/L	P value	LDH IU/L	P value
Sham	3.86 ± 0.3*	0.001	2.93 ± 0.4*	0.001
Sham + GA	2.91 ± 0.5*	0.001	1.85 ± 0.3*	0.001
Ctr	12.10 ± 0.6^$^	0.001	7.00 ± 0.7^$^	0.001
GA 2 h	6.90 ± 0.6*	0.001	3.88 ± 0.2*	0.01
GA 2 W	5.98 ± 1.4*	0.01	3.65 ± 0.6*	0.01
GA 4 W	7.85 ± 0.9*	0.01	4.08 ± 0.2*	0.01
GA + Rep	11.45 ± 0.8^$^	0.001	6.78 ± 0.6^$^	0.001

*CK* creatine kinase; *LDH* lactate dehydrogenase, *Ctr* control, *GA 2 h* gum arabic infusion 2 h before sacrifice, *GA 2 W* gum arabic administration for 2 weeks, *GA 4 W* gum arabic administration for 4 weeks.

*P < 0.01, **P < 0.001 compared to control and ^$^P < 0.001 comparede to sham and sham + GA.

### Role of cytokines in GA-mediated protection of the heart from ischemia–reperfusion injury

**Figure 4:**
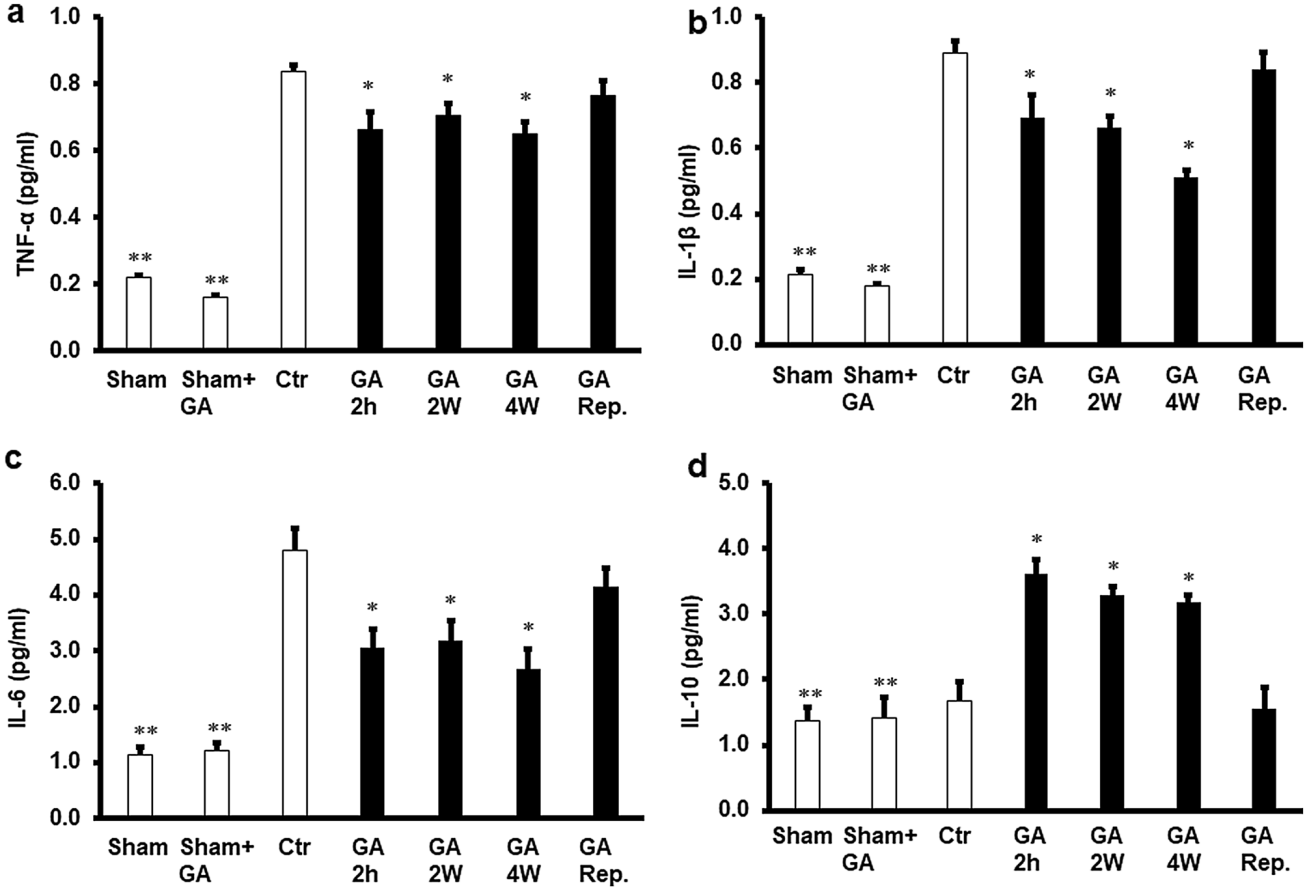
Pro-inflammatory and anti-inflammatory cytokine levels in the cardiac muscle samples after GA for 2 weeks, 4 weeks, or 2 h before sacrifice or at reperfusion compared to those in the control group (n = 4). GA decreased the TNF-α protein levels (**a**), IL-1 protein levels (**b**), the IL-6 protein levels (**c**) and had no effect on the anti-inflammatory cytokine IL-10 (**d**) protein levels. *Ctr* control, *GA* 2 h Gum Arabic infusion 2 h before sacrifice, *GA 2 W* Gum Arabic administration for 2 weeks, *GA 4 W* Gum Arabic administration for 4 weeks, *GA Rep*. Gum Arabic infusion at reperfusion. *P < 0.05 compared to the respective controls and **P < 0.01 compared to controls and other treatments.

To study the modulatory effects of cytokines on I/R injury and their possible relation to the protection provided by GA, the levels of TNF-α, IL-1β, IL-6 and IL-10 were measured in the cardiomyocyte lysate and in the coronary effluent by ELISA. Exposure of the heart to GA for 4 weeks, 2 weeks or 2 hours before heart isolation resulted in a remarkable decrease in the protein levels of TNF-α, IL-1β and IL-6 compared to the untreated controls (P < 0.05). Moreover, cytokine levels were significantly lower (P < 0.01) in sham and GA treated sham control groups than in untreated controls and all other GA treated groups (Fig. 4a,b,c). There were no significant differences in TNF-α, IL-1β, IL-6 protein levels between the treatments with GA for 4 weeks, 2 weeks or 2 hours before the heart isolation (Fig. 4). There were also no significant difference in the levels of TNF-α, IL-1β and IL-6 between the sham and GA treated sham groups.

To evaluate the potential anti-inflammatory effects of GA, the levels of IL-10 were estimated using ELISA. The administration of GA for 4 weeks, 2 weeks or 2 hours before heart isolation resulted in a significant (P < 0.01) increase in IL-10 levels compared to untreated control (Fig. 4d). This cytokine levels were significantly higher than that in sham and GA treated sham less than the other GA treated groups (Fig. 4d). Interestingly, administration of GA at the time of reperfusion did not impart a significant effect on TNF-α, IL-1β, IL-6 or IL-10 levels (Fig. 4a,b,c,d) compared to untreated controls.

### Expression levels of oxidants and antioxidants with GA treatment

**Figure 5:**
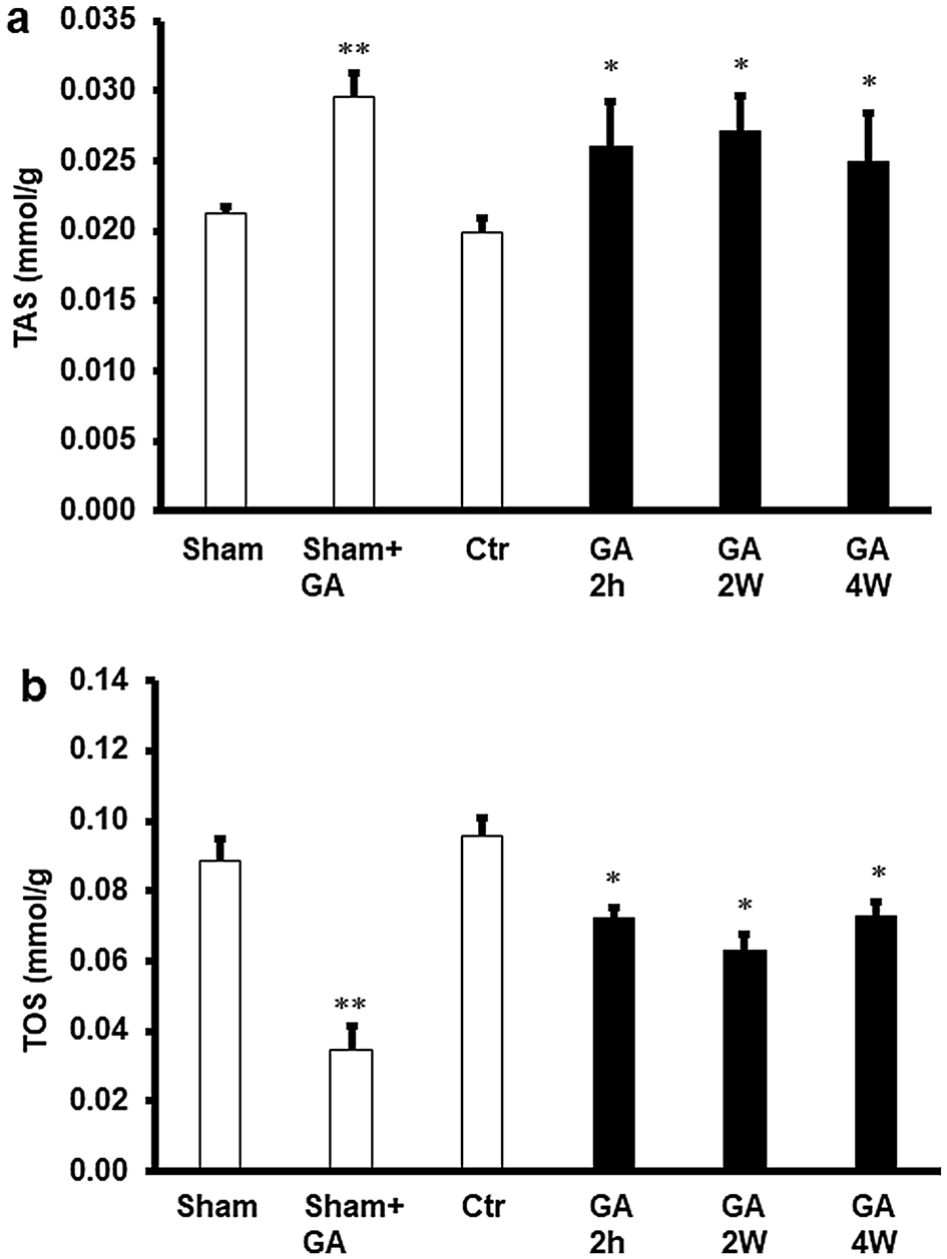
Estimation of oxidative stress in rat hearts. (**a**) Total oxidant and (**b**) total antioxidant levels in cardiac muscle samples after GA administration for 2 weeks, 4 weeks, or 2 h before sacrifice at the end of treatments period and before I/R procedure compared to the levels in the control group (n = 4). *Ctr* control, *GA 2 h* Gum Arabic infusion 2 h before sacrifice, *GA 2 W* Gum Arabic administration for 2 weeks, *GA 4 W* Gum Arabic administration for 4 weeks, *GA Rep*. Gum Arabic infusion at reperfusion. *P < 0.01 compared to the respective controls and **P < 0.01 compared to controls and vehicle treated sham.

**Figure 6:**
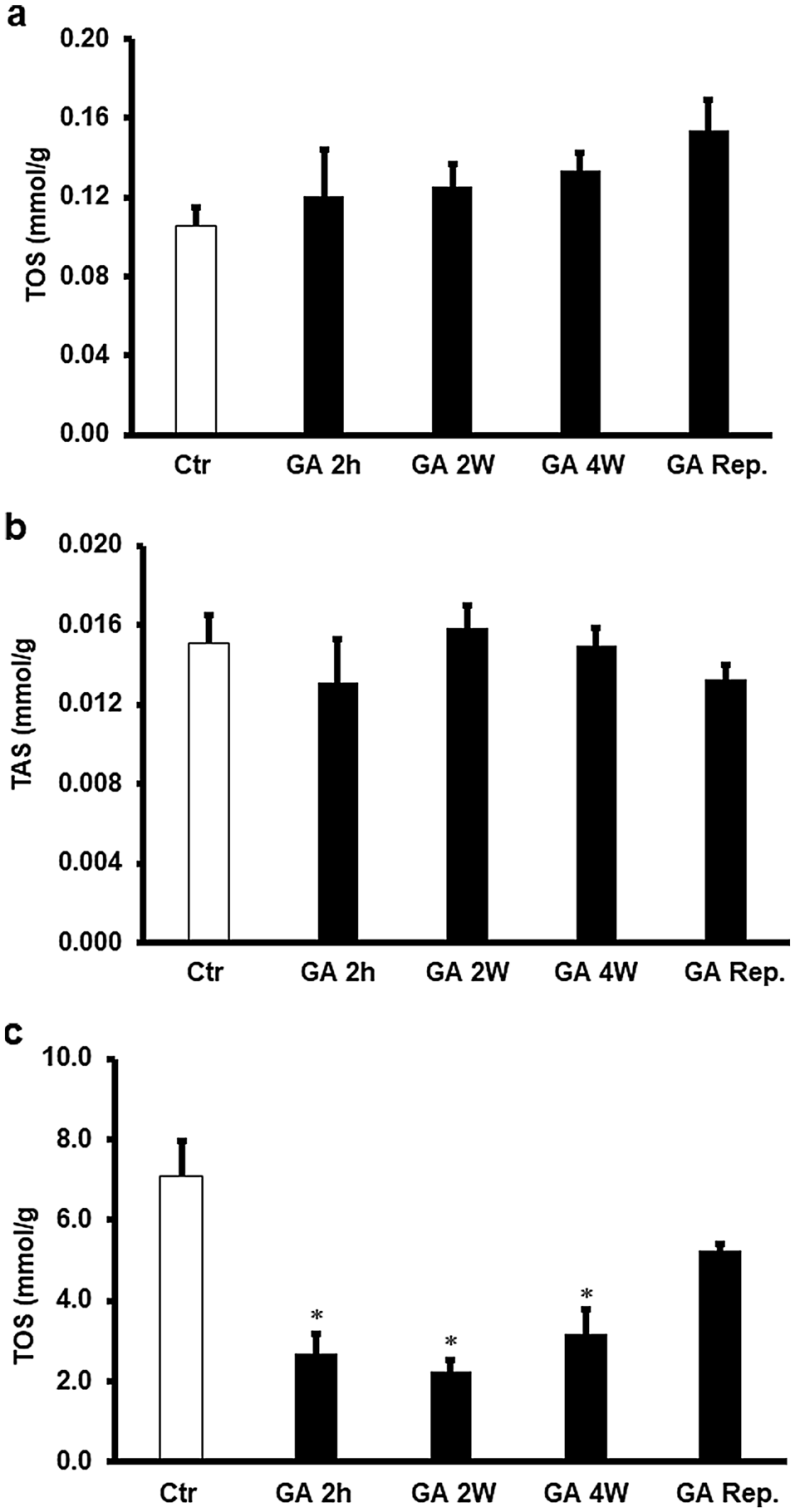
Estimation of oxidative stress in rat hearts. (**a**) Total oxidant and (**b**) total antioxidant levels in cardiac muscle samples after GA administration for 2 weeks, 4 weeks, or 2 h before sacrifice or at reperfusion compared to the levels in the control group (n = 4). (**c**) Total oxidants levels in the effluent samples after GA administration for 2 weeks, 4 weeks, or 2 h before sacrifice or at reperfusion compared to the levels in the control group. *Ctr* control, *GA 2 h* Gum Arabic infusion 2 h before sacrifice, *GA 2 W* Gum Arabic administration for 2 weeks, *GA 4 W* Gum Arabic administration for 4 weeks. *P < 0.01 compared to the respective controls.

GA treatments for 4 weeks, 2 weeks or 2 hours before heart isolation resulted in a significant increase in TAS levels and a significant decrease in TOS levels (P < 0.01; Fig. 5a,b) when they were evaluated in hearts isolated from the rats at the end of GA treatment compared to the respective untreated controls. However, TAS levels in the sham group were similar to that in control group, administration of GA to sham hearts significantly increaseed the TAS levels compared to the vehicle treated sham and untreated control groups (P < 0.01 and P < 0.01, respectively) (Fig. 5a). TOS levels in the sham group were similar to that in the untreated control group, and administration of GA to sham hearts resulted in less ROS levels than in the vehicle treated sham and untreated control groups (P < 0.01) (Fig. 5b). Surprizingly, I/R intervension abolished the significant differences in the levels of TOS or TAS shown in Fig. 5 compared to those of untreated control hearts (Fig. 6a,b). However, the TAS levels in the coronary effluent collected at the end of reperfusion were below the detection level. The TOS levels were significantly reduced by GA treatment for 4 weeks, 2 weeks or 2 hours before sacrifice compared to the respective controls (P < 0.01). This decrease in TOS levels was not observed when GA was infused at reperfusion (Fig. 6c).

**Figure 7:**
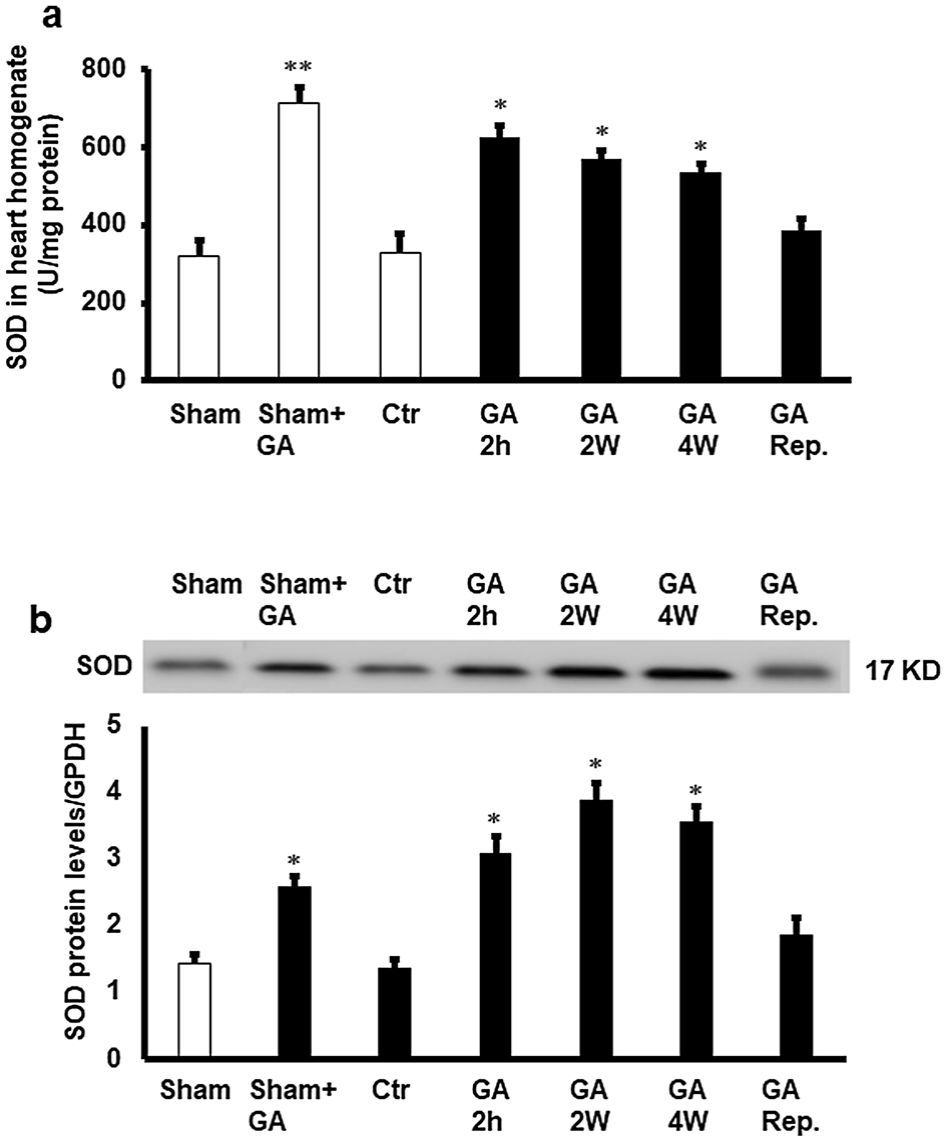
Estimation of antioxidant enzyme SOD. (**a**) SOD protein activity the cardiomyocytes (n = 4). (a) SOD protein levels in the cardiomyocytes (n = 4). Top: Western blot showing the protein levels of SOD corrected to GAPDH. Bottom: SOD protein levels corrected to GAPDH. SOD in cardiac muscle samples after GA administration for 2 weeks, 4 weeks, or 2 h before sacrifice or at reperfusion compared to the levels in the control group (n = 4). *Ctr* control, *GA 2 h* Gum Arabic infusion 2 h before sacrifice, *GA* 2 W Gum Arabic administration for 2 weeks, *GA 4 W* Gum Arabic administration for 4 weeks, *GA Rep*. Gum Arabic infusion at reperfusion. *P < 0.01 compared to control and **P < 0.01 compared to control and vehicle treated sham. Full length gels were included in supplementary figure (Fig. 7S).

The effect of GA treatment on the antioxidant enzyme SOD was studied. Ischemia/reperfusion decreased the cardiomyocyte SOD levels compared to controls (P < 0.01), which was significantly (P < 0.01) increased by GA treatment for 4 weeks, 2 weeks and 2 hours in consensus with reduced TAS levels. This increase in SOD levels was not observed when GA was infused at reperfusion (Fig. 7). Although the SOD levels in the sham group were not significantly different from that in untreated control group, administration of GA to sham hearts with GA resulted in higher levels of SOD than in sham and untreated control groups (P < 0.01) (Fig. 7).

## Discussion

### Significance of Gum Arabic mediated heart protection against I/R injury

**Figure 8:**
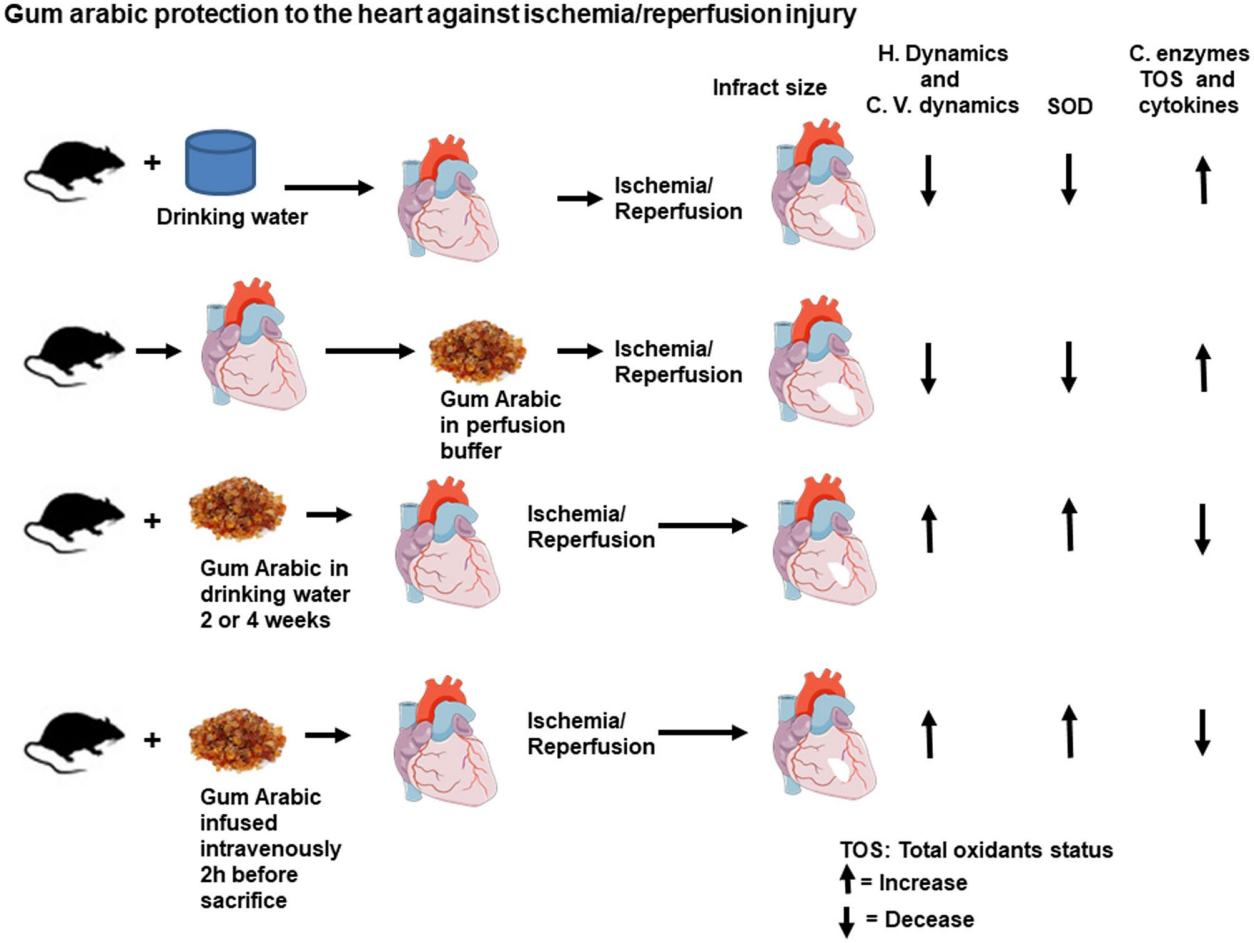
Schematic representation of the protective effects of GA shown in this study (n = 8 for hemodynamics and (n = 4) for infarct size, antioxidants effects and anti-inflammatory effects). *Ctr* control, *H. dynamics* hemodynamics, *C. dynamics* cardiovascular dynamics, *C. enzymes* cardiac enzymes, *SOD* superoxide dismutase, *TOS* total oxidants, *GA 2 h* Gum Arabic infusion 2 h before sacrifice, *GA 2 W* Gum Arabic administration for 2 weeks, *GA 4 W* Gum Arabic administration for 4 weeks, *GA Rep*. Gum Arabic infusion at reperfusion.

This study demonstrates the protective effects of GA against heart I/R injury. Treatment of the heart with GA for 4 weeks, 2 weeks or 2 hours before heart isolation resulted in a substantial improvement in the left ventricular and coronary vascular dynamics. This treatment also protected the heart from I/R injury and decreased cardiac enzymes levels and infarct size. In contrast, the infusion of GA at the time of reperfusion did not protect the heart from I/R injury (Fig. 8). To the best of our knowledge this is the first study evaluating the therapeutic effects of GA in the protection of the heart against I/R injury, therefore the literature regardingthe role of GA in protectingthe heart against I/R injury is lacking. However, treatment of renal failure patients with GA mitigated the renal failure-associated increased blood pressure^17,19^. The report of these protectiveeffects on the the heart highlighted the potential effects of this molecule in the protection of the heart. Gum Arabic was also reported to reduce bood pressure in diabetic patients and patients with kidney failure^17,27^. The same mechanisms could be responsible in the protection reported in this study, however, more experimental evidence is required. Moreover, our study reported a role for GA in dreasesing free radicals. Our findings are in line with studies that showed antioxidant activities for GA^16,28^. In the current study, GA showed potent protection to the heart by ameliorating oxidative stress. Our results are in line with the results reported by Abd-Allah and his co-workers^29^. Moreover, GA treamnet increased the antioxidant SOD enzyme level which was shown to play a crucial role in the protection of the heart from I/R injury^30^. Although not directly related to our study, studies in kidney failure illustrated a decrease in cell injury following GA treatment^21,31^. The protection seen in our study could be related to the antioxidant^29^ and/or the anti-inflammatory activities of GA^32^ which have beendemonstrated previously. The lack of protection when GA was infused at reperfusion could be because the length of GA infusion period was not sufficient to allow neutralization of free radicals and pro-inflammatory cytokines. However, our expetrmental setting does not allow evaluation of the interaction of other organs in this protection, this effect may exist in in vivo studies.

### Pathways that may be involved in GA-mediated heart protection from I/R injury

The use of GA and its lack of toxic effects is well established in the literature^19,28,33^. Although it was postulated that GA protects organs and tissues through its anti-inflammatory and antioxidant effects, the mechanism of this protection are still unknown. Even though residence of macrophages was reported in the literature, their presence in the isolated heart is unlikely^34,35^. Indeed, the heart is cabable of producing pro-inflammatory cytokines when challenged with ischemia^36^. These pro-inflammatory cytokines exacerbate the cardiomyocye injury and their neutralization could be one of the protective pathways of heart from ischemia. Our study uses isolated perfused heart which completely exclude the presence of resident macrophages. Therefore, the source of the pro-inflammatory cytokines are the stressed cardiomyocytes. This study evaluated the potential involvement of pro-inflammatory cytokines in protective effects of GA on heart against I/R. Several studies have shown that myocardial ischemic insult promotes formation of pro-inflammatory cytokines, which contribute to cardiac dysfunction, cardiomyocyte necrosis and apoptosis^37–39^. Treatment of rat heart with GA prior to reperfusion showed a significant reduction in these pro-inflammatory cytokine levels. We previously reported the decrease of TNF-α levels is a key factor in the protection of the heart from I/R injury^38–41^. The neutralization of the TNF-α and decrease of its levels by GA protected from I/R injury in other organs^16^. However, the lack of observable reduction in pro-inflammatory cytokines at reperfusion in the current study could be due to the short duration of exposure to GA or possibly due to exposure to a smaller dose. Lowering the IL-1β level was proven to be essential in the protection of the heart against I/R injury and was shown to decrease apoptosis of cardiac myocytes^42,43^. IL-6 was also proven to be involved in the suppression of heart function and in the exacerbation of cardiovascular diseases^44^. It has been consistenetly detected in patients with stable or unstable angina and MI^23^. Lowering circulating IL-6 levels crucial for heart protection^42^. The results of this study are consistent with theses previous studies.. Furthermore, Our data showed that treatment of the heart with GA significantly prevented the increase of pro-inflammatory cytokine levels as reported previously by Nemmar et al.^45^. On the other hand, the anti-inflammatory cytokine; IL-10 was reported to be crucial for heart protection against I/R injury^46^, nevertheless in this study, treatment with GA increased IL-10 protein levels. This finding is in agreement with Ali et al.^16^, who reported a significant increase in IL-10 protein levels as a consequence of GA treatment. However, this was not the case when GA was infused at reperfusion which could be explained by the short time of GA availability or possibly a higher dose is required. However, GA was proven to produce a direct anti-inflammatory action^47^ and also the soluble fibers in the GA were reported to show anti-inflammatory action in IL-10 deficient mice^48^, results which support our findings.

This study investigated the effects of GA on oxidant and antioxidant levels before and after I/R injury (Fig. 8) and its effect on the oxidative stress biomarker SOD. Although some studies suggested a dose-dependent antioxidant activity of GA^49^, others reported no antioxidant activity for it in I/R injury^50^. Our recent study did not show significant differences in the levels of TOS and TAS in cardiomyocytes compared to the respective untreated controls after I/R procedure. However, administration of GA neutralized TOS in the cardiomyocyte as shown by the decrease of their levels in the coronary effluent. Treatment with GA revoked the evident decrease in SOD created by the I/R in the untreated control groups. These findings specifically indicate a neutralizing and antioxidant effects of GA on oxidative stress. The neutralizing and antioxidant activity of GA were reported by other researchers in other experimental settings^21,45^. The explanation for this phenomenon could be related to the antioxidant effects of GA, which neutralize ROS produced by the cardiomyocytes and ameliorate their possible autocrine and paracrine detrimental effects as shown in the GA treated sham group in the current study. The antioxidant activity of GA was additionally confirmed in this study by its increase of SOD protein levels which were decreased by I/R (Fig. 7). Our findings are in consensus with the antioxidant activity of GA which was reported in some other studies using different experimental protocols^16,21,28,33,51^. This study proves that GA protects the heart by a pathway employing its antioxidant and anti-inflammatory activities. This pathway ultimately decreased the apoptotic enzyme levels and production of pro-inflammtory cytokines.

A limitation of this study is that the best procedure for the evaluaqtion of the infract size is to present it as a percentage of risk area. However, in this study the infarct size was corrected to the left ventricle area. To solve this problem, we standardized the occlusion point in all hearts and the infarct size data were normalized to the LV area. Furthermore, corroboration of the infarct size with the cardiac enzyme data further confirm the infarct size and mitigate this limitation.

## Conclusions

GA protects the heart from I/R injury if it is administered before the induction of ischemia and reperfusion, but no such protective effects are imparted if it is administered at the restenosis of the coronary atery. The GA-mediated heart protection from the injury is regulated through alleviation of oxidants by upregulating antioxidants, which inhibit injurious effects of pro-inflammatory cytokines.

## Supplementary Information

Supplementary Figures.

## Data Availability

The data used to support the findings of this study are included within the article. Data is also available on request to corresponding author.
