# Supplementary figures and images for: Gum Arabic protects the rat heart from ischemia/reperfusion injury through anti-inflammatory and antioxidant pathways
Source: Sci Rep. 2022 Oct 14;12:17235. doi: 10.1038/s41598-022-22097-0 (PMC9568585; doi:10.1038/s41598-022-22097-0)

Figure 3S

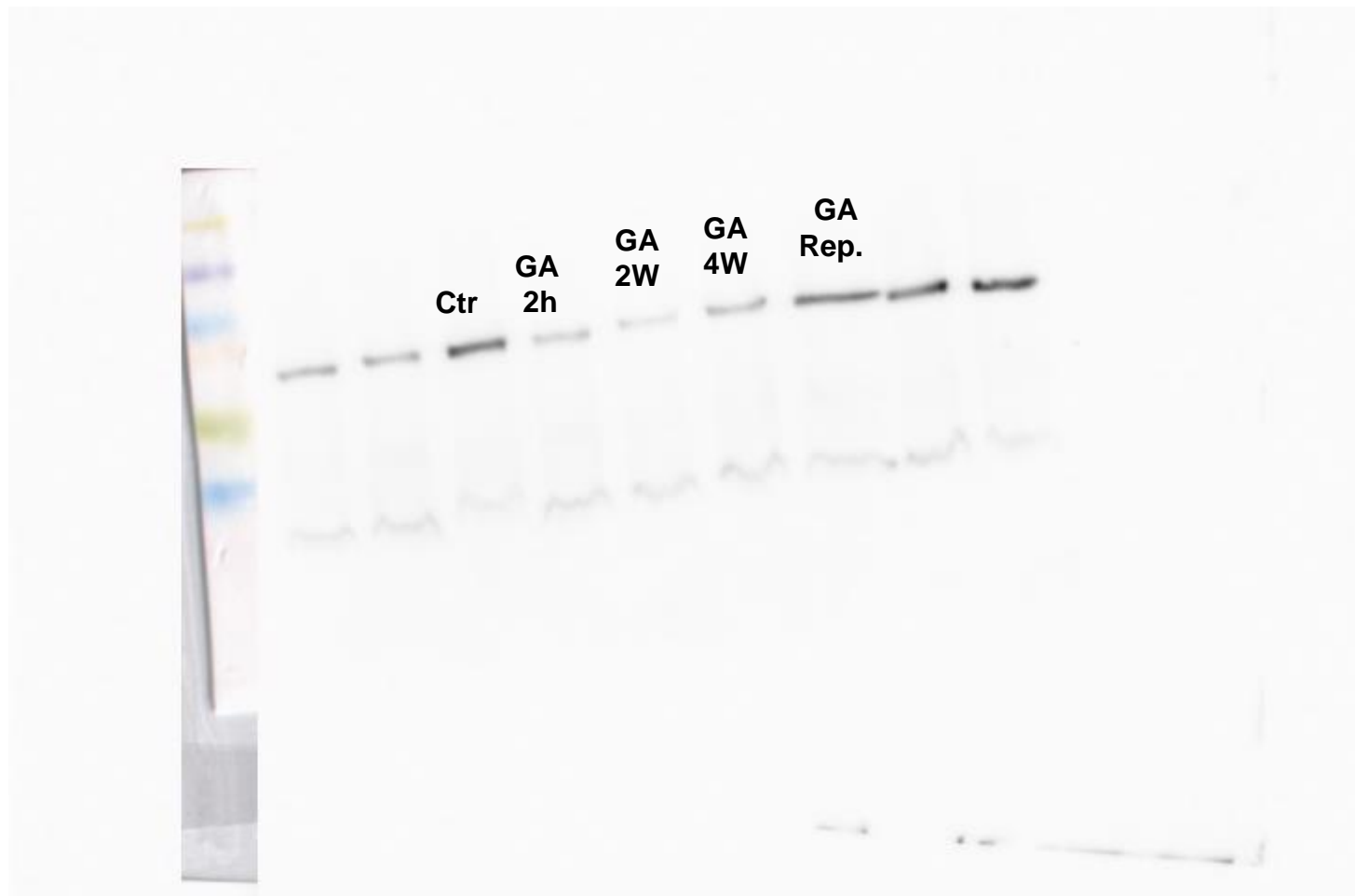

Figure 7S1

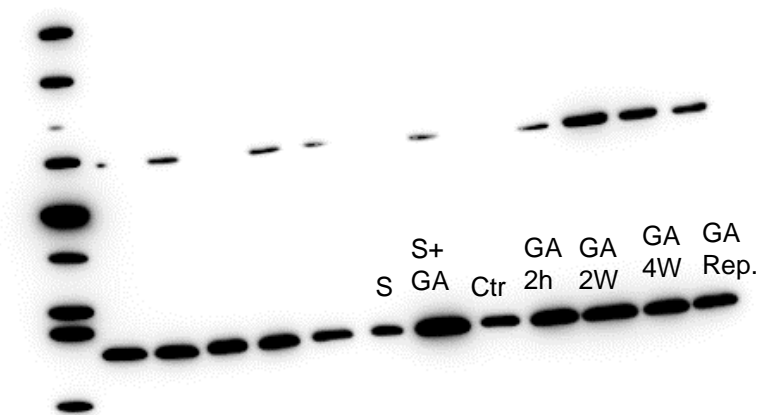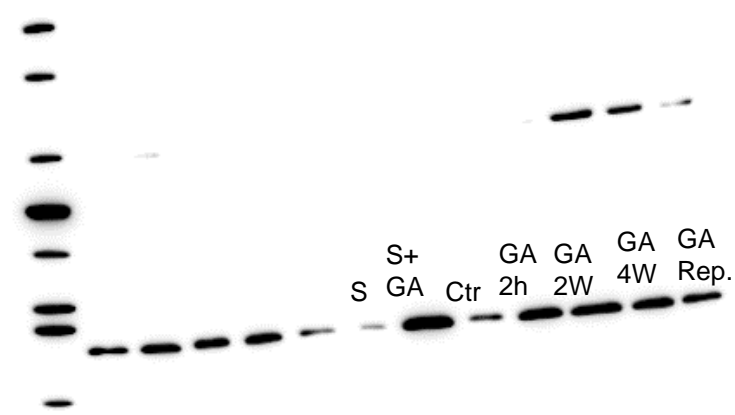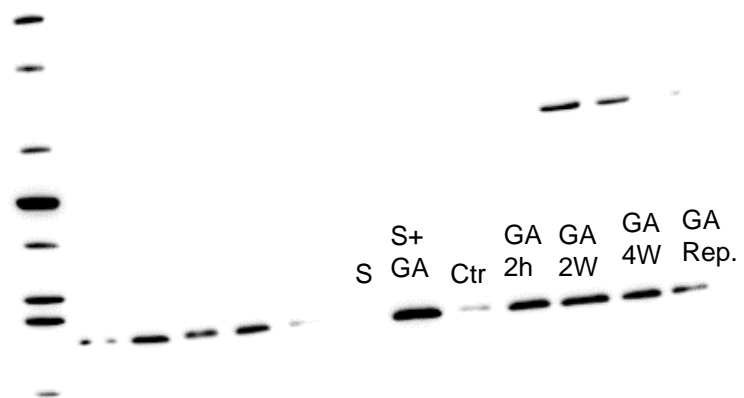

Supplement: Supplementary file 1 — Supplementary Figures. [file 41598_2022_22097_MOESM1_ESM.pdf]
